# Wildlife in U.S. Cities: Managing Unwanted Animals

**DOI:** 10.3390/ani5040401

**Published:** 2015-11-11

**Authors:** John Hadidian

**Affiliations:** The Humane Society of the United States, 2100 L St. NW, Washington, DC, USA; E-Mail: jhadidian@humanesociety.org; Tel.: +1-301-258-3144; Fax: +1-301-258-3080

**Keywords:** urban wildlife, pest, nuisance wildlife, human-wildlife conflict, animal welfare, humane wildlife control

## Abstract

**Simple Summary:**

Wild animals are increasingly adapting to living in urbanizing environments, even as urban living has become the dominant human life style. This leads to greater opportunities to experience and enjoy wildlife, but also to increases in the kind and frequency of human-wildlife conflicts. Conflicts occur not only with species deemed to be perennial pests or nuisances, but situationally and episodically with others that are valued and esteemed. Regardless of how we view wild animals with whom we have conflicts, all deserve moral consideration and humane treatment. Issues in coexisting with urban wildlife are reviewed here.

**Abstract:**

Conflicts between people and wild animals in cities are undoubtedly as old as urban living itself. In the United States it is only of late, however, that many of the species now found in cities have come to live there. The increasing kind and number of human-wildlife conflicts in urbanizing environments makes it a priority that effective and humane means of conflict resolution be found. The urban public wants conflicts with wildlife resolved humanely, but needs to know what the alternative management approaches are, and what ethical standards should guide their use. This paper examines contemporary urban wildlife control in the United States with a focus on the moral concerns this raises. Much of the future for urban wildlife will depend on reform in governance, but much as well will depend on cultural changes that promote greater respect and understanding for wild animals and the biotic communities of which they and we are both a part.

## 1. Introduction

Although wild animals have certainly lived in cities for as long as humans have, much less is documented about their lives than is desirable. We can be fairly sure that early cities were not hospitable to most species of wildlife and that those hardy enough to survive in them were likely to have been regarded as pests, as many still are. With a few exceptions (e.g., [1]), the popular literature on urban natural history is a post-World War II phenomenon [2,3,4]. Attention from scientists and wildlife professionals, again with few exceptions (e.g., [5]), comes even later [6]. In addition, if the emergence of a discipline can be measured by the appearance of its first texts [7,8], then the field of urban wildlife has barely started. Although Adams [9] correctly derives the field from a preceding focus on wildlife conservation and game management, urban wildlife may be better regarded today as belonging in urban ecology, another emerging discipline. The study of urban wildlife suffers from the lack of focus, rigor and definition typical of nascent disciplines. This applies especially to its governance, most notably in the area of human-wildlife conflicts. The purpose of this paper is to review the status of urban wildlife in the United States, especially with an eye towards human-wildlife conflicts and some of the moral concerns these raise. I will focus primarily on the United States because I know it best and because it may be best documented for the subjects addressed here, although there are obvious parallels with issues on a global scale [10]. For the most part this treatment excludes discussion of the commensal rodents, since they typically engage different administrative, bureaucratic and professional sectors than do native species.

There are a number of challenges in undertaking this effort. What “urban” itself means can be ambiguous, describing entities that range from metropolises with millions of residents to small aggregations with only a few tens of thousands. The peri- and ex-urban associates of urban centers can be highly variable as well, made up of complex landscape mosaics. There is a gradient from city core to outlying wildlands along which ecological change occurs [11,12] but “typical” urban human-wildlife conflicts still are found, in part because the social identity of residents remains urban. Urban species are highly varied, from near ubiquitous rats (*Rattus norvegicus*) and raccoons (*Procyon lotor*) to highly local javelinas (*Pecari tajacu*) in the desert southwest and elk (*Cervus canadensis*) in cities of the mountainous West. Urban areas are culturally diverse, with their varied populaces bringing different knowledge, attitudes, values and beliefs into consideration of the human dimensions of wildlife. Clearly, the physical and social environments for urban wildlife are dynamic, complex, and heterogeneous.

I will only touch on issues of relevance here because they are far more complex than can be addressed in a single paper. Much of the information available to us on urban wildlife is anecdotal or incompletely assembled and I will rely on examples to help clarify some of the important points raised. I will first attempt to place urban wildlife into context as “unwanted”, next set the social environment for conflict, then address the nature of conflicts in brief and describe the various stakeholders to conflicts and the approaches that are taken to control wild animals in cities. I will then focus on the humaneness of urban wildlife management, as well as the management contexts in which urban wildlife control does, as well as might, occur.

## 2. What Does “Unwanted” Urban Wildlife Mean?

Wild animals living in cities are variously identified as residents, colonizers, invaders, ferals, and overabundant, and more specifically characterized as inquilines, synanthropes and commensals. The term “unwanted” is commonly understood when applied to stray and abandoned cats and dogs [13], but far less employed with wild animals than terms such as “pest”, “nuisance”, and “vermin”. A pest is a human construct, the status of which can change as people’s perceptions and values change and about which our knowledge is imperfect [14,15]. The term “nuisance” as well has more to do with human perception than any characteristics inherent to animals or their ecology [16]. Both terms are implicitly negative, although not so much as “vermin”, “deteriogens” [17], or “sky carp”, which Ankney [18] reports as used by some to describe urban Canada geese (*Branta canadensis*). Terms have meanings and the failure to operationalize them can be harmful [16], potentially desensitizing the public as well as introducing vagueness and subjectivity into scientific and professional discourse. Well-defined terms are needed to help set law and policy, making a better lexicon for urban wildlife an important objective. The use of unwanted follows the Confucian dictate that “If names be not correct, language is not in accordance with the nature of things” [19] by placing the onus on how people feel about animals, rather than on an animal’s particular behavior, natural history or ecology.

### Those Dirty Raccoons

“How a once revered furbearer became reduced to cockroach status” reads the subtitle to this one page account in a well-known outdoors magazine [20]. Deriving material from an article in a prominent Chicago newspaper and interviews with state wildlife agency personnel, the article goes on to mention how a woman was “hissed at” by a pair of young raccoons who had invaded her home and how raccoon numbers were skyrocketing as the trapping industry suffered from low pelt prices. The article portrays a fairly common theme in popular and uninformed writing about urban wildlife: populations are exploding and animals are increasingly becoming threats; things used to be better and we ought to go back to the way we were.

## 3. The Social Environment for Urban Wildlife

Studies of the wildlife preferences of urbanites consistently indicate strong liking for certain groups such as songbirds and a disliking for animals that are feared (such as snakes) or commonly associated with causing problems (such as mice) [21,22,23,24]. No other wildlife-related activity engages more Americans than backyard bird feeding or birdwatching, with a majority of birdwatchers coming from urban areas [25]. Kellert [23] found that urban Americans showed a strong tendency to favor moralistic and humanistic stances toward wild animals, while rural Americans valued wildlife more for practical and material (utilitarian) uses. The rise of non-utilitarian attitudes was associated with the demographic shift from rural to urban living in the United States [26]. More recent research has reframed these observations by identifying behavior shifts from doministic to mutualistic value orientations occurring as societies move from industrial to post-industrial status [27,28,29,30]. This is relevant when considering how urban Americans feel about the management of urban wildlife, especially along its nonlethal to lethal axis. In general, urbanites do not support lethal management of wild animals [23,31,32]. This tendency, however, appears dependent on the type of risk and threat people face (or think they face) as well as whether or not individuals have directly been involved in conflicts themselves.

More than half of the residents of the Chicago metro area found it acceptable to destroy wildlife that is spreading disease to people, while only slightly more than a quarter approved of lethal control if there were safety risks to their pets [24]. Coluccy *et al.* [33] found a majority of Missourians enjoyed seeing Canada geese, but that those who experienced direct impacts in the form of property damage were likely to view them negatively and be more supportive of lethal management. Similar sentiments extend to white-tailed deer (*Odocoileus virginianus*) [34,35,36], indicating that the negative encounters individuals experience with wild animals are important in determining tolerance for individual species. This raises concerns that these might generalize into broader antipathies for all wildlife [37,38]. The public may be encouraged to look on animals as human opponents in “Nature Wars”, whether these are being fought with insects as traditional antagonists [39] or with deer, geese and other higher vertebrates as emerging combatants [40]. Calls for a “Backyard Battle Plan” [41] or mobilization of the public for “Squirrel Wars” [42] are made somewhat tongue-in-cheek of course, but they also may help create an environment that undergirds calls for lethal action or activities that disregard concerns about humaneness against species judged to be problems.

Lindsey and Adams [43] (p. 279) conclude that most urbanites operate in an “intellectual and experiential vacuum” with regard to encounters with wild animals. Inside that vacuum it is important to understand how people perceive risk, given that such perception is likely to drive behavior [44,45]. One dilemma with urban wildlife control is that perceived risks or those based on fear or misunderstanding may be disproportionately influential in driving behavior. Many urbanites may also still hold to a simple belief that wild animals do not “belong” in cities and have no right to be there, or would certainly be better off if they could be moved to their “natural” habitat.

### Urban Coyotes

Coyotes have recently colonized many major metropolitan areas in the U.S. [46]. Conflicts with coyotes as they first move into an area can be quite different from those occurring after they become established residents, both because the behavior of the animals is changed and because the novelty of their being present has passed. Coyotes are territorial and a pair that is not causing problems (such as killing small pets left outdoors) may keep other coyotes who might do so away. Once people become familiar with the presence of coyotes they ideally would learn not to leave pets outside unattended, not feed pets outdoors and engage in better trash management and other cultural practices to minimize attractants. Aversive conditioning through citizen hazing programs could help shape the behavior of resident coyotes to head off potential problems. Rather than mount eradication campaigns as sometimes called for in the initial alarm at their appearance, people and coyotes both benefit from learning what the rules of coexisting are while settling into generally nonconflict relationships.

## 4. The Nature of Conflicts with Urban Wildlife

Conflicts with urban wildlife can be visualized as events (raccoons scatter trash left at the curb) or processes (deer grazing impacts the vegetation in a municipal park), and sometimes both. Conflicts have spatial and temporal distributions, and their timing, periodicity, sequencing, and intensity are factors influencing their nature and expression as well as control, stabilization or resolution. Conflicts tend to be situationally and contextually distinctive, involving animals who may only be causing a perceived problem, be wrongly identified as causing a problem, actually be causing a problem or, as sometimes codified in state regulations, simply be animals that “may” cause problems (e.g. [47]). The impact of actual problems can range from trivial to severe. Problem-causing animals may be rare, common, or numerous; increasingly the term “overabundant” is used to justify management, despite the ambiguities associated with that concept [48,49].

The problems wild animals cause may affect people, property, pets, wildlife, or public resources such as woodlots or water supplies. Conover *et al.* [50] and Conover [51] found physical injury, illness, vehicle collisions and property damage were most cited in metropolitan areas as reasons justifying control. Aircraft strikes are an important public safety concern and animals around airports may be lethally controlled in preemptive measures [52]. Protecting biodiversity, preventing environmental degradation and/or species extinctions, predation on and disease transmission to pets and other preferred species, and even indirect effects such as alteration of nutrient cycling have also been raised as general concerns with problem wildlife, urban species included [53].

Beyond conflicts affecting individuals lie those that can involve neighborhoods, communities, and at times entire municipalities. To address conflicts at the larger landscape level, open public processes and collaborative, community-based management concepts are increasingly advocated [15,54,55,56]. These are not so much intended to completely resolve disputes between stakeholders as to allow for more open engagement and transparency through processes relatively new in wildlife management [57]. Warburton and Norton [58] argue that many conflicts involving wildlife damage involve such polarized opinion that different interest groups may not even agree as to what the problems are. These “wicked” problems [59] do not have technical solutions and may involve deeply rooted conflicts. Such conflicts may best be addressed by professionals trained in conflict intervention theory and practice [60]. Implicit in such approaches is the idea that human-wildlife conflicts can be as much or sometimes more about conflicts between people over wildlife than conflicts between animals and people themselves.

The human dimensions of human-wildlife conflict are complex and extend well beyond the concepts addressed above into areas only recently gaining fuller attention. The role of better means of communication is increasingly emphasized as establishing not only clear understanding of what different sides mean when speaking to an issue, but what they perceive and weigh as risks, not to mention adhere to when cognizing such fundamental constructs as “nature”. Perception of risk, disproportionate responses and social influences are invoked as important features of human-wildlife conflicts [61] while methodological approaches taken from other disciplines, such as the concept of fault lines from political science [62], are leading to both a better understanding of the nature of human-wildlife conflicts as well as the practices needed to address them.

### Raccoons in the Trash

A homeowner leaving trash at the curb in plastic bags is dismayed to find that raccoons are ripping the bags open and scattering it overnight. After calls to municipal agencies asking for their intervention prove unhelpful, a referral to a local company specializing in wildlife trapping meets with immediate response and service for a fee in capturing the offending animals. The service provider finishes the work in a few days and collects the fee; the homeowner either does not care or perhaps does not want to know what is done with the raccoons, but state regulations mandate euthanasia or release on site, so it is clear they are killed. The service provider could have simply advised the homeowner to put the trash out on the morning of collection, not the night before, or to place the trash in a raccoon resistant dumpster, but this would be of no financial benefit to the company. The trapping does solve the problem, at least for a few weeks until other raccoons reoccupy the vacant habitat.

## 5. Who Controls Urban Wildlife?

Much less information is available on the kind and number of animals controlled in cities, the type and volume of problems, and the identity of those who engage in wildlife control than would be desirable. Conover [51] surveyed 1000 households randomly selected from 100 of the largest metropolitan areas in the United States and reported 57 percent of residents admitting to problems with wild animals in the previous year. Miller *et al.* [24] reported similar (58%) findings from a regional survey of the Chicago metropolitan area. Conover [51] further reported that 69 percent of metropolitan residents surveyed were involved in managing wildlife around their homes, with 52 percent of their control efforts being unsuccessful. The Miller *et al.* [24] study reported slightly more (71%) who attempted their own solutions, nearly a third of these using household chemicals. In most states, private property owners or their agents can typically control nuisance wildlife on their land by any legal means, including trapping and translocation or euthanasia, although some states may require that owners or their agents obtain a permit to do so. Live-trapping and translocation of animals such as squirrels and raccoons by private individuals as a “humane” solution to dealing with problem wildlife is unquestionably quite common, although accurate estimates of this activity are hard to come by.

Resources and services available to the public in resolving conflicts with urban wildlife can come from local animal shelters and humane societies, nature centers, wildlife rehabilitators, nonprofit organizations, state and federal agencies and private businesses. The U.S. Department of Agriculture’s Wildlife Services contracts with a few state wildlife agencies to run a toll-free wildlife advice line and may participate in fee-for-service contracting with municipalities and private entities in projects to cull deer, capture and kill Canada geese and trap and kill beaver and coyotes, as well as others [63]. Wildlife Services also plays a major role at airports implementing Bird Aircraft Strike Hazard (BASH) plans [64]. Municipal animal care and control agencies (shelters and local humane societies) may handle considerable numbers of wild animals through providing loan of live traps and then either relocating or euthanizing trapped animals as a service to the community. Some provide telephone advice and most will directly respond to wild animals in the living space of homes or wildlife in distress. These activities are typically unreported in literature.

Langenau [65] predicted that a significant industry would become established around urban wildlife control and this has been proven with the proliferation and growth of private businesses providing for-fee services [66,67]. One of the first and subsequently largest franchisers of nuisance wildlife businesses is Critter Control, recently acquired by pest industry giant Rollins. A trade organization, the Nuisance Wildlife Control Association (NWCOA) now affiliates with the larger National Pest Management Association (NPMA), bringing further organizational consolidation to the industry. State wildlife agencies claim the same regulatory responsibility and authority for wildlife in cities as they do elsewhere [68,69] and thus bear the principal responsibility for oversight of the private industry sector. Wide variation exists in the type and quality of statutory and regulatory provisions, however [70,71]. Hadidian *et al.* [72] surveyed the fifty states and District of Columbia on ten categories of oversight, ranging from requiring licenses to standards for humane handling and care and assigned a plus one if statutes and/or regulations existed to address the category and zero if not. The resulting national score for the states had a mean of 2.16. Only one state has published information from annual reports submitted by commercial businesses, this being data from a nine year period in Illinois [73], noting that a total of 483,608 wild animals were handled, with a majority (180, 324) being raccoons, of which slightly more than 127,000 were killed. Adams [69] surveyed state wildlife agencies and found fewer than one percent of staff biologists occupying positions that focused on urban wildlife, leading him to conclude that an “infrastructure” for urban wildlife was lacking. This can be attributed in part to the novelty of wildlife (or at least many species of wild animals) on the urban scene and in part to the overwhelming emphasis state and federal agencies have traditionally placed on game species or agricultural pests, leading to bureaucratic and academic infrastructures that have been “captured” by special interests [74].

### Municipal Animal Control

Pittsburgh Animal Control, as one example of a municipal animal shelter with a tradition of loaning traps to the public, euthanized an average of more than 1500 raccoons/year between 2011 and 2014 [75]. The agency also provides services by picking up trapped animals and bringing them to the shelter for euthanasia, which appears to have led to some of the individuals borrowing traps to document the number of animals trapped on their property and post challenges online to others to do better.

## 6. How is Urban Wildlife Controlled?

Urban wildlife control involves the use of many of the same tools and techniques employed for wildlife elsewhere, except that lethal practices may be somewhat more limited and constrained. The use of explosives, fumigation, and diseases, for example, would be largely prohibited in cities, while shooting, trapping and poisoning might be restrained or curtailed. This would not include, however, the massive amounts of poisons used on rats and mice by individuals, businesses and municipal agencies. The indiscriminate and over- use of rodenticides has identifiable welfare and conservation consequences, with secondary and nontarget exposures resulting in the deaths of protected and valued species [76]. Concern over the use of the most dangerous rodenticides may not trump public demand for control, as some products remain widely employed although known to be inhumane [77,78] even among those who support the use of toxicants [53]. Poisoning of other species appears uncommon and is illegal, with the exception of avicides used to poison birds, primarily pigeons, but also species such as gulls, blackbirds and crows as well [79,80]. Anecdotal reports of people leaving bowls of antifreeze outside to kill wild or domestic animals they feel are problems exist, but the extent of this practice and others like it is undetermined.

A popular mode of wildlife control for some in the public is to trap and translocate smaller animals such as raccoons and squirrels [81]. Once thought to be both humane as well as an ideal solution to resolving problems with locally abundant animals [82], this approach is increasingly being recognized as problematic. Among the reasons cited for this are possible welfare compromise for translocated animals [83], transmission of disease [84] unacceptable costs [85] and uncertainty over whether the approach resolves the conflicts it was intended to resolve [32]. Trapping and euthanasia of problem animals is equally controversial and probably more commonly practiced than trapping and translocation by commercial businesses [73]. Commercial pest control companies typically are not permitted access to euthanasia drugs nor to the training available to technicians at animal shelters, restricting their use of appropriate euthanasia techniques [86]. The use of unstudied, untested and unapproved chemicals in wildlife euthanasia is particularly problematic, as in the example of acetone (Methyl ketone) [86] to kill skunks via thoracic injection. Drowning is used by both private individuals and some commercial businesses as a euthanasia technique, despite strong evidence that it is inhumane [87,88], even though this was contested by some wildlife professionals [89]. The complex issue of necessity also engages the debate over euthanasia. Existing published standards for animal euthanasia mainly address procedures for use on animals in production, companion animals or animals in controlled environments, such as zoos [90,91]. Standards for wild animals in the U.S. do not yet take into account factors such as the stress experienced merely being in the presence of humans, as well as the impact associated with handling, transport and holding prior to an animal’s actually being put to death.

State wildlife agencies typically promote hunting as a preferred means of population control for species such as deer and geese where not a threat to public safety. Hunting is also often limited in urban areas because of public opposition [92]. To have a meaningful impact on urban wildlife damage, hunting would have to reasonably ensure that a large enough portion of the problem-causing population can be removed to achieve a desired benefit, usually reduction of some type or amount of damage. Conover [93] argues that hunting could also be beneficial if it changed the behavior of animals in ways that led to reduced damage, but this remains to be confirmed for urban wildlife. Culling is a specialized lethal practice, again usually involving deer and geese, which typically engages hired specialists or volunteer agents. Deer are typically baited and killed by sharpshooters or captured under drop nets and killed with captive bolt devices [94]. Geese may be shot, but more typically are rounded up when in the annual molt by herding into pens and either dispatched on site using carbon dioxide chambers or transported to poultry facilities and killed in the same manner as commercially processed birds. Culling of deer has been argued as more humane than hunting because of lower wounding rates, especially when compared to bow and arrow hunting [95].

Trapping is especially problematic for many animal welfare and rights groups [96], but staunchly defended by others who argue it has a role in controlling problem-causing animals [97,98]. Traps of many sorts are used in urban wildlife control, including box and cage traps, “body-gripping” devices such as steel-jawed leghold traps, “enclosed” foothold traps, foot and body snares, and killing traps, including Conibear-type body crushing traps, neck snares, and drowning sets for animals such as beaver [99]. Some states, as well as individual counties and municipalities, ban all body-gripping and body-crushing traps, while others restrict their use to documented public health and safety priorities [99]. International efforts to improve animal welfare have prompted responses from trapping interests to create Best Management Practices (BMP’s), but to date no BMP’s have been issued for urban wildlife [100].

Although strategies to control reproduction in wild animals are popular with the public and have been fairly well researched during the past half century, the majority of the concepts explored have not proceeded beyond test stages [101,102]. Currently only one product (OvoControl-P, registered for use with pigeons) is commercially available in the United States, while immunocontraceptive vaccines for use primarily on deer, horses, and a wide variety of zoo animals are in use under special or restricted permits or allowances. While fertility control undoubtedly has a promising future as a humane means of population limitation, this will best be realized only when orally effective compounds become available. Current approaches have significant technical, logistical and political hurdles to overcome [103,104] and may be stymied for some time by having to pass through onerous developmental and testing stages. This is how many technologies develop however, although usually with better funding and political support.

There are many different kinds of nonlethal tools and strategies used in urban wildlife control, including repellents, frightening devices and physical exclusion. Home supply and hardware store shelves testify to the variety of products commercially available, and home remedies (such as mothballs to force animals to move out of an attic) abound, with most of dubious value and suspect from a welfare perspective. In any given case nonlethal strategies may work to satisfaction, with exclusion generally providing the most lasting effects. Any nonlethal strategy, however, can fail if animals are highly motivated, are particularly numerous, habituate to aversive stimuli, or can defeat physical barriers. Monitoring and renewed response is needed and especially important when employing nonlethal strategies. Nonlethal tools and practices can have adverse welfare consequences for wild animals [105] and this should always be recognized and evaluated as a program component.

Gates *et al.* [106] and Griffin *et al.* [107] describe a novel approach for wild animals that use human structures. This evict-exclude-reunite strategy displaces problem-causing animals while allowing for family units (usually mothers with dependent offspring) to remain together within the bounds of a known home range. This means displaced animals simply need to locate another den site, while still able to make use of other well-known resources, such as food. This approach has been criticized as likely to just shift problems onto other homeowners by the reasoning that animals with a proclivity for the use of structures will make these first choices in the relocation process [108]. This remains to be better determined, but the considerable number and type of dens used, for example, by urban raccoons [109] suggests otherwise.

### Nuisance Wildlife Disposal

Cea [110] provides advice in a trade journal suggesting wildlife control operators tell customers as little as possible about disposition of trapped animals, other than that they would be “relocated”, followed by further tips on how to use the large 25 gallon buckets that swimming pool chlorine comes in as killing chambers. These air-tight containers kill by suffocation, which he notes “…can be presented as a very humane way to dispose of wildlife as the animal simply goes to sleep” [110] (p. 11). In addition to humaneness, transparency and consumer protection are important elements of urban wildlife control that to date have been little addressed openly.

## 7. Humaneness and Urban Wildlife Control

Humaneness is sometimes said to be merely the subjective reaction of observers to the pain and suffering experienced by others, while at other times is characterized as an objective assessment of pain and suffering itself [105,111]. In line with the latter, Kirkwood *et al.* [112] proposed four welfare measures—the number of animals affected, the cause and nature of the harm, the duration of harm and the capacity of the animals to suffer—that contribute to an empirical construct for humaneness. Developments in welfare assessments for animals, wildlife included, then provide a means of implementing humane approaches. Assessment is an integral process in animal welfare science that seeks to systematically evaluate welfare impacts and the means by which better welfare can be achieved [113]. Hickling [114] advocates use of a “decision cube” in assessing welfare as a part of control programs, where axes of low to high impacts, certainty of benefits from control, and low to high extent of suffering can be used to rank programs as more or less humane. Following a similar tact, Sharp and Saunders [105,115] propose a matrix assessment model that examines both nonlethal and lethal interventions along a scale of severity and duration, allowing individuals with different interests and expertise to rank the humaneness of both nonlethal and lethal actions along a scale of severity and duration. This approach offers a practical way to integrate both expert and lay opinions about lethal and nonlethal components of control while remaining based in a framework that recognizes two significant welfare concerns—severity and duration.

Regardless of such objective approaches, subjective reactions to killing, as opposed to non-lethal techniques, also determine views on humaneness. The public is concerned about the humane treatment of wildlife, even when perceived as nuisances [66], and this probably drives the preference of most nuisance wildlife control companies to advertise their services as “humane”. Companies make this claim even when they kill animals who need not necessarily die, justifying it by their use of killing techniques that meet conventional standards for providing a humane death. This issue of necessity in killing can be compared with that of “unnecessary suffering” as an animal welfare concern [116], with both in need of further explication. The use and misuse of the term “humane” should be examined more thoroughly, particularly with respect to urban wildlife.

### Relative Humaneness

In 1984 the New Jersey legislature enacted a ban on all body gripping or leghold traps. In the spring of 2015 the New Jersey Fish and Game Commission, an appointed body that passes recommendations to the New Jersey Division of Fish and Wildlife, proposed that the state allow the use of three trap devices commonly known as enclosed foothold traps on the grounds that these were different enough from the banned devices to warrant their exclusion from the legislated ban. This difference can only be explained by appeal to the argument that this class of devices is more selective in trapping manually dexterous animals such as raccoons and opossums, only occasionally captures domestic cats and some mustelids, and effectively will not capture domestic dogs and other canids [117,118,119]. Opponents of the devices argue that the “humaneness” argument is based solely on this selectivity, while the powerful coil springs and steel bar that pin the victim’s limb do not differentiate these devices from other banned traps. They argue that the appeal to “relative” humaneness is an attempt to circumvent the issue that the devices are not at all humane for the animals that do get trapped by them.

## 8. Managing Unwanted Urban Wildlife

No consensus approach to urban wildlife control has yet emerged, and it is unlikely one will soon, given the many different private and public interests involved and the complex practical as well as moral issues that should be engaged. This does not mean that a framework for dealing with this activity should not be initiated, however. The discussion below focuses on some elements of what might be called criteria, elements, guidelines or principles for urban wildlife control. At present they would largely serve heuristic purposes, but initial guidance has at least been drawn up for commercial operators (e.g. [120]) and these could form the basis for more broadly adopted playbooks for many public and private interests.

The point of entry when considering any wildlife control program is whether control is necessary in the first place [121,122]. To those who assume it is a rightful prerogative of humans to do as they wish with wild animals this question would seem irrelevant, but for those who feel that how wild animals are treated is a morally relevant concern, an ethic for urban wildlife becomes important [123]. As Lynn [124] notes, ethics can represent a “blizzard of concepts”, and it would be beyond the scope of this review to try to address that field here. An ethical framework for urban wildlife, however, will provide a basis for addressing moral dilemmas, help in making moral choices, and help improve reasoning processes [125]. Such a framework should include consideration for individual animals as well as populations and species and their habitats. Leopold’s [126] articulation of a land ethic established the basis for consideration of habitat (the land) as an ethically relevant construct and placed respect and concern for land directly in opposition to the utilitarian and consumptive use orientation of traditional development interests. Dorney [127] somewhat optimistically predicted that an ecology-systems approach would dominate decision-making in development planning rather than traditional approaches based on economic and political expediency, leading him to articulate an “ethical triad” for urban ecologists that consisted of reverence for life, reverence for land and reverence for diversity. Beatley [128] outlined a series of ethical principles as a moral foundation in guiding the development process, but it appears that these and a concern for wildlife and habitat still are not part of what Dorney [127] characterized as the “frame of reference” that development interests embrace. As guidance for how we as individuals might behave, Lockwood [129] proposes a minimum ethic for insects that calls for refraining from causing them harm when our own interests are not, or only trivially are, being affected. Samways [130] extends this concept to argue that the protection of habitat is an essential prerequisite in the protection of populations and species, laying groundwork for advocating that the biodiversity of cities be valued and conserved, as is increasingly becoming the case [131]. Thus, a number of concepts are on the table that may productively be applied to the varied contexts and situations in which urban wildlife is found.

Decision-making in wildlife damage management has traditionally focused on areas such as economy, efficacy, selectivity and safety as first order concerns [132], and it is only relatively recently that calls have been made for animal welfare to be considered an equal priority [121]. While rodent control has long been the separate focus of public health and sanitation agencies [133], it does provide a model in Integrated Pest Management (IPM) concepts that could contribute to our thinking about managing conflicts with other vertebrate species as well [134,135]. IPM seeks to integrate science, technology and policy in programs that aim to achieve “continual improvement” [136] and tacitly acknowledge that eradication will be unfeasible. This would be especially true for a majority of urban wildlife, as no program could meet the specific criteria required to be successful in eradication [137] while being acceptable to a majority of the public. If eradication is not feasible, the focus of control then shifts to establishing acceptable outcomes through the setting of clear objectives [14,15]. An emerging general set of management principles for wildlife damage that follows an IPM-like series of decision-making steps begins to address this need [78,122,138,139]. The following summarize the steps involved:

- The need to act must be clear. (justification)
- Any benefits sought must be realistic. (achievability)
- The methods to be employed must be able to achieve benefits. (effectiveness)
- The approach must be targeted to the problem-causing individuals. (specifity)
- The methods used must be the most humane available. (welfare priority)
- The consequences of actions must be amenable to evaluation. (monitoring)
- The benefits achieved must be maintained. (follow-up)

This leaves us to account finally for the varied situations and contexts within which conflicts between humans and wild animals occur and control is implemented. In this, there is a strong argument for pluralistic approaches in ethical guidance [58,127,140]. Eggleston *et al.* [140], as one example, advocate a framework for wildlife damage management that in its broader context is based on Principalism—a pluralistic approach that does not allow any one set of principles to dominate but uses “mid-level” constructs such as non-malfeasance to improve ethical consideration.

### Canada Geese on the Anacostia River

In compliance with the National Environmental Policy Act (NEPA) the U.S. National Park Service published a final environmental impact statement in 2014 for the Anacostia Watershed in Washington, DC, USA, addressing the management of a restored 100 acres of wetlands along one of the nation’s more degraded waterways [141]. The preferred alternative calls for a combination of wetland management (most of which would be deferred because of expense) together with the annual culling of 40 to 60 percent of the “resident” geese found within the vegetation restoration areas. This would be preceded by some amount of destruction of eggs and nests as these could be located. The process is to be open-ended and is deemed necessary because “resident” geese remain year-long in the area and subject the wetlands to more grazing pressure than would a migratory population. Such “resident” geese are subject to permissive depredation permitting authorized by the U.S. Fish and Wildlife Service which views them essentially as a non-native component of the biotic community. Critics have argued that the Park Service’s plan is neither reasonable nor humane, since the effective designation of some geese as “non-native” is arbitrary and the effort to protect only a tiny fraction of a much larger and highly degraded waterway is ecologically unrealistic.

## 9. Discussion

Urban wildlife control encompasses a wide variety of human decisions and actions. Moving a turtle out of the road is urban wildlife control, as is rounding up and killing geese when they are in molt or so young as to never have flown at all. There is a growing need for both practical as well as moral guidance on human-wildlife interactions in urbanizing environments, both for the sake of the animals and people as well. The future for humans seems decidedly to be an urban one where both the kind and frequency of interactions with wildlife is likely to increase. New species of animals will colonize cities, and those already there will adapt to the environmental challenges they face, some to become in all likelihood entirely new species, as has already happened with plants [142]. This will give us a very different moral lens through which to look at conventional ideas about rarity in the natural world and an opportunity to appreciate that cities in their completeness can be biotic communities able to be visualized in entirely new ways [143].

It is not entirely clear what trajectory human-wildlife interactions will follow in cities of the future. Today’s urbanites are emerging from a period of unfamiliarity with many of the species now common in cities and there is a possibility that the public will simply adjust to wild animals as familiar and accepted. Kellert [23] suggested that education would be a critical part of the future of urban wildlife, and it certainly would be important in reconnecting urbanites with the natural world [128,144]. Van Herzele *et al.* [62] stress the role of communication in bridging social divisions. For all parties engaged in urban wildlife, a minimum ethic at least could take form and begin with Lockwood’s dictate that we ought to refrain from killing or causing pain when it would have no or only trivial consequences for ourselves. Such an ethic would not be enforceable, but would have to be taught. 

For those providing governance, as well as advice or commercial services, other challenges exist. If Adams [69] is correct and the infrastructure for urban wildlife is missing, then creating one becomes a priority. For now the commercial arm of urban wildlife control appears to be following a traditional pest industry template in which profitability dictates the type and tempo of services, and wild animals increasingly come to be devalued and treated with questionable methods. State wildlife agencies could provide more resources and guidance, but this would require both an administrative commitment and an ideological shift which seems unlikely at this time. An alternative model of wildlife control could emerge from traditional animal care agencies and local humane societies who already have the suitable infrastructure, trained and experienced personnel, and organizational ethos to deal humanely with unwanted animals. Such services would be truly community based. Alternatively, in the proper statutory and regulatory environment, small consulting entities that subcontract work according to specific requirements, as envisioned by Dorney [145] for environmental management, might be possible. A biophilic city concept currently being advanced [146] explores ways of directly connecting urban populaces with nature, both through biophilic design concepts [147] as well as educational and experiential focus on fostering understanding and respect for the natural world [144]

The future for urban wildlife will depend not only on reforms in governance such as these or others not currently foreseen, but as well on cultural change and acceptance that a humane future is not only desirable, but feasible as well.

## 10. Conclusions

Although wild animals have been a part of urban living since cities began, it is only quite recently that attention has turned to urban wildlife as a field of interest unto itself. Wild animals in cities are often viewed as problematical but undoubtedly are also beneficial, for their aesthetic values, as indicators of environmental quality and as key to appreciating nature in the city. Full exploration of human-wildlife relationships in urbanizing contexts has only just begun and many challenges exist in determining what is needed as proper management and governance. Our approaches to urban wildlife should be governed by an overarching ethic that holds these animals to be worthy of moral consideration and humane treatment, whether they are pigeons on the streets of New York city or macaques taking to the roofs in Mumbai.
